# Management of Calcaneal Cysts in the Pediatric Population: A Review

**DOI:** 10.5435/JAAOSGlobal-D-22-00248

**Published:** 2023-03-10

**Authors:** Caleb Gottlich, John C. Fisher, Dominic Campano, Michel Diab

**Affiliations:** From Department of Orthopedic Surgery, Texas Tech University Health Sciences Center, Lubbock, TX.

## Abstract

Cysts of the bone are a common pathology that, although benign, are frequently treated because of their tendency to compromise the integrity of affected bone. Two common entities are unicameral bone cysts and aneurysmal bone cysts. Although these are two distinct pathologies, they are treated similarly and thus will be discussed in tandem. The optimal treatment of calcaneal bone cysts in pediatric patients has long been debated among orthopaedic surgeons because of the relatively small number of cases and varied results within the literature. Currently, there are three lines of thought regarding treatment: observation, injection, and surgical intervention. When considering which course of treatment is best for an individual patient, the surgeon must consider the fracture risk without treatment, the risk of complications with treatment, and the recurrence rate with each treatment approach. There are limited data on pediatric calcaneal cysts specifically. Still, there are much data concerning simple bone cysts of long bones in the pediatric population and calcaneal cysts in the adult population. Because of the lack of literature on the subject, there is a need for a review of the available literature and a consensus on the approach to treating calcaneal cysts in the pediatric population.

Cysts of the bone are a common pathology that, although benign, are frequently treated because of their tendency to compromise the integrity of affected bone. These lesions can occur consequential to other forms of bone pathology or arise de novo. When discussing benign cysts of the bone, the two entities in question are unicameral bone cysts (UBCs) and aneurysmal bone cysts (ABCs). Although these are two distinct pathologies, they are treated similarly and thus will be discussed in tandem.

Bone cysts and neoplasms occur in the calcaneus at a rate of 2% to 3%.^1,2^ UBCs most commonly occur in children and affect male patients twice as often as female patients.^3^ ABCs are also common in children but affect male patients only slightly more than female patients.^4^ Diagnosis of UBCs and ABCs is most often accomplished through radiographs. Occasionally, CT and MRI may be used if radiographs are not diagnostic. The diagnosis is made histologically in cases where there is a concern for more aggressive pathologies.

Unicameral bone cysts are the most common pathology of the calcaneus, with estimates in the literature of UBCs accounting for around 40% of all cystic tumor-like lesions of the calcaneus.^1^ Initially, the pathologic process was believed to be an inflammatory process with fluid from cyst aspirate containing several osteodegrading compounds, such as interleukins and prostaglandins. Treatment was focused on reducing inflammation with steroid injections into the lesion.^5^ This has proven to be beneficial effects but often requires multiple injections and generates inconsistent results. Cysts of the calcaneus are particularly recalcitrant to steroid injections compared with cysts occurring elsewhere throughout the skeleton.^5^ Other proposed methods of UBC formation are erroneous bone formation and ossification, or osteological response to trauma or failure of hematoma resorption.^6^ Finally, there has been speculation recently that there is a genetic contribution to UBC formation based on the appearance of mirror image UBCs in two separate case reports of monozygotic twins.^2,7–9^

ABCs are estimated to account for as many as one-quarter of the lesions in the calcaneus, with about two of every three arising de novo and the rest the resulting sequelae of other oncologic processes.^1,10,11^ One theory that has strong support is that venous obstruction is responsible for the formation and progression of these cysts through increased intraosseous pressure.^5,12,13^ A biopsy, which is often necessary to differentiate from other pathologies such as a chondroblastoma, will demonstrate an expansive capillary network lined by endothelial cells with or without remnants from a preexisting lesion dependent on if the lesion is secondary to another process or arising de novo.^13,14^ Treatment success rates are determined by the activity level of the cyst, with active lesions requiring more aggressive margin resection and bone grafting.^15^

The optimal treatment of calcaneal bone cysts in pediatric patients has long been debated among orthopaedic surgeons because of the relatively small number of cases and varied results within the literature. Currently, there are three lines of thought regarding treatment: observation with serial radiographs, injection with steroids or bone marrow, and surgical intervention. Although each of these treatment groups has numerous variations, we will discuss them collectively in this article. When considering which course of treatment is best for an individual patient, the surgeon must consider the fracture risk without treatment, the risk of complications with treatment, and the recurrence rate with each treatment approach. There are limited data on pediatric calcaneal cysts specifically. Still, there are much data concerning simple bone cysts of long bones in the pediatric population and calcaneal cysts in the adult population. Because of the lack of literature on the subject, there is a need for a review of the available literature and a consensus on the approach to treating calcaneal cysts in the pediatric population.

## Surgeon's Consideration

The calcaneus is the sixth most common anatomic location for cysts to manifest in children.^16^ Currently, there are no universally accepted criteria to guide a surgeon's management of simple bone cysts. The concern is that fracturing of the calcaneus can result in impaired mobility, persistent pain, and activity restriction.^5,17^ Because of this, even when asymptomatic, many orthopaedic surgeons opt to prophylactically address bone cysts to mitigate potential fracture and ensuing bone collapse.^5^ Pogoda et al.^18^ attempted to define a critical size at which surgery should be favored over conservative management. They described critical size as a cyst that fills the calcaneus in the coronary plane from the lateral to the medial cortex and occupies greater than 30% of the anterior to posterior distance in a transverse CT scan. Patients were also stratified based on whether they were symptomatic on presentation. Based on these criteria, 37 patients were treated with observation, 29 had cysts that did not qualify as critical in size, and eight were critical in size but were asymptomatic.^18^ The small cysts showed no progression or pathologic fracture to the adjacent bone at 1 year of follow-up. The eight cysts, which were critical in size but asymptomatic, did not progress or fracture and were followed for 36 months. The surgical group of 10 patients experienced no complications and had no cyst recurrence. Several other studies of bone cysts involving the long bones of pediatric patients have been published with cited risk factors of cortical thickness of less than 1 mm, a cyst index (cyst area/diaphysis diameter) of >5, and an active phase UBC, defined as growing adjacent to the epiphyseal plate.^19,20^

In addition to the risk of fracture, orthopaedic surgeons must also consider the risk of recurrence and the complication risk of a procedure. The literature shows that although most simple bone cysts are not progressing with observation, they are also not spontaneously resolving.^16,21^ This contrasts with surgical intervention in which treatment was often curative and recurrence was infrequent.^16,17,22,^ There have been a very few mentions of serious complications secondary to surgical intervention in the literature, most of which are from single case reports.^5,16,18,23,24^ Because of the relatively low risk involving surgical intervention and the high probability of success, many orthopaedic surgeons are treating simple bone cysts surgically. Because there is no published consensus on the management of these patients, the risks and benefits of each treatment modality should be considered on a case-by-case basis, including healing rate, risk of recurrence, and risk for fracture. These aspects are considered in further detail below.

## Treatment Modalities

### Observation

Conservative management is preferred in the pediatric and adult populations whenever possible. Observation is commonly used as the primary treatment modality regarding simple bone cysts. The main concern with observation is that the cyst could continue to progress, potentially leading to a pathologic fracture. When this occurs, there is a risk for long-term complications including, but not limited to, compromised bone or joint, which could give way to chronic pain. Pogoda et al.^18^ treated 40 of 50 (80%) of their patients over 10 years with observation and experienced no fractures based on their parameters of critical size and pain discussed above. Another study by Donaldson and Wright^25^ showed no pathologic fractures in the 24 patients they followed for a mean of 7 years. Finally, in a study published in the Journal of Foot and Ankle Surgery, there were no complications of pathologic fractures reported in the 86 nonsurgical patients followed for 3 years.^16^ Despite there being no pathologic fractures in the observation group in these three studies, none showed healing of the cysts, defined as a reduction in size greater than 50%.^16^ This means that the theoretical risk of pathologic fracture that exists initially persists and likely increases over the patient's life.

Importantly, these studies, except for Donaldson and Wright, did not look at the pediatric population specifically. In addition, although none of these studies showed pathologic fracture in the observation arm, it is worth noting that there are numerous case reports discussing fractures that have occurred secondary to simple bone cysts. It is unknown if these case reports would be within the critical size and symptomatology parameters set forth by Pogoda et al. and thus warrant surgical treatment.

### Steroid/Bone Marrow Injection

Injection with steroids was an early treatment approach for simple bone cysts due to the theory that cysts were the result of an inflammatory reaction. Although this theory did not stand up as the sole cause of simple bone cysts, there is a notable amount of data looking at the healing rates of injected cysts, the recurrence rate, and complications for simple bone cysts in general. The data are widely varied on whether steroid injection or bone marrow provides better results, with some studies showing that steroid injection is superior and others showing no difference.^6,16,17,26,32^ Cho et al.^6^ showed that steroid and bone marrow injection had similar success rates, but steroid injection was associated with a higher recurrence rate in bone cysts, irrespective of location. Conversely, Chang et al.^26^ showed no difference between steroid and bone marrow injection for simple bone cysts independent of location. Finally, in a systematic review of the available literature, Zhao et al.^27^ could not identify a statistical difference between steroid and bone marrow injection. Because of the inconsistent data regarding the efficacy of steroid versus bone marrow injection, we will consider injection broadly as a treatment modality. The theoretical risks of injection to the calcaneus, as with any injection, include infection and iatrogenic damage to the surrounding structures. However, despite these theoretical risks, a very few complications have been reported with the injection group.^16,26^ The healing rates of cysts injected with steroids or bone marrow are widely varied, with some studies showing no healing of the cyst and others showing rates as high as 66%.^16,17^ Zhao et al.,^27^ in their systematic review, cited success rates of <50% for both steroid and bone marrow injection at 2-year follow-up for long bone simple bone cysts. Differences in frequency, number, and the amount injected are likely to account for the contradictory data. In addition, most studies showed that more than one injection was required before an effect, if any, was seen.^27,28^ In a study of 11 patients over 7 years at two institutions, Glaser et al. found that steroid injection alone did not resolve simple bone cysts and that surgery on these patients did provide radiographic healing. Glaser suggests that although injection of simple bone cysts in other areas of the body, such as long bones, has provided positive results, injection of the calcaneus is uniquely refractory, and surgical intervention is generally required.^17^ Injections are an alternative to observation in nonsurgical management with few risks or complications. Still, success rates are widely varied, and some data show that it may not be an ideal treatment modality for calcaneal bone cysts.

### Surgical Management

Numerous studies discuss surgical management of calcaneal UBCs in the literature. Current surgical options include open curettage with or without bone augmentation, cannulated screw decompression, and more recently, endoscopic curettage. The different treatment options have varying success rates, but each surgical method provided superior healing rates to observation or injection, demonstrating efficacy rates from 85% to 100% depending on the approach.^16^ Regarding complications from surgery, no postoperative infections, loss of function, or nerve damage was reported in any studies we reviewed. Saraph et al.^22^ reported one case of nine patients who had irritation at the screw insertion site, which required early removal, but no other complication was noted.

A systematic review conducted by Levy et al. reported that pain was improved with all surgical approaches by 47% to 100%. Several aspects were included in the review; noteworthy that open curettage with and without augmentation (both allograft and autograft) had statistically notable improvements in pain, although no statistical difference existed between the two forms of augmentation.^16^ Furthermore, in their systematic review, Levy et al.^16^ showed that autografting resulted in higher healing rates than the allograft. Also discussed was endoscopic curettage with allograft augmentation, the newest approach included in the review, which has been the topic of numerous subsequent studies. These studies investigated operating time, length of stay in the hospital, overall healing, reduction in pain, and length of time to return to full activities.^23,24,29,30^ Operating time and length of stay in the hospital varied between articles, with some showing no difference in the operating time and others showing increased operating times with endoscopic surgery.^24,30^ The success rate was 100%, with no reported recurrences or failure to heal among the 44 patients, although the sample size is relatively small.^23,24,29,30^ Also noteworthy is the absence of reported complications and markedly improved pain.

In 2015, Shirai et al demonstrated that treatment of a simple bone cyst could be reliably treated in the calcaneus with fenestration of the cyst, curretage, and insertion of a cannulated ceramic pin coated with hyaluronic acid. This was shown to be most efficacious in calcanual cysts and was proffered as a viable treatment option due to the minimally invasive nature of the intervention as well as the reliability in which it led to cyst resolution. This has been demonstrated by other authors with recommendation of cannulated hydroxyapatite screws be used as they do not need to be removed.^31,33^

Finally, in the two studies reporting on the return time to full activities, endoscopic curettage with allograft augmentation allowed for a return to full activities in half the time of open curettage (6.5 weeks vs. 14.5 weeks).^24,29^ These studies demonstrate that endoscopic curettage offers a minimally invasive approach with notable improvements in pain, healing, and return to activities with no reported complications.

## Summary

The management of cysts of the calcaneus has long been controversial among orthopaedic surgeons, especially in the pediatric population. Diagnosis is often incidental, found in asymptomatic patients. Observation carries the risk of subjecting patients to a pathologic fracture and the complications of pathologic fracture. On the other hand, surgical management has high cure rates and low recurrence but carries the physical, financial, and emotional risks common to surgical management. In addition, there is a concern that surgical management may be unnecessary and overly aggressive as small cysts rarely progress.^18,25^ In 2004, Pogoda et al.^18^ posed a critical size, which they defined as a cyst that spans 100% of the intracalcaneal cross-section in the coronary place and at least 30% in the sagittal plane. In addition, they only operated on patients whose cysts met the critical size and were symptomatic. Following these rules, they reported no pathologic fractures in patients whose cysts did not meet the critical size. In our literature review, we could not find any case reports of pathologic fractures in patients who did not meet the critical size of a cyst. Based on these findings, we suggest that surgical management be reserved for patients who meet the Pogoda criteria.

When surgical management is deemed necessary, our review shows that endoscopic approaches are preferred over open curettage and cannulated screw decompression due to the comparable outcomes and reduced exposure. This is contingent on the surgeon's ability to reduce the time under anesthesia and time in the operating room. Endoscopic techniques allowed for high radiographic healing rates, up to 100%, with no recurrence noted.^23,24,29,30^ In addition, endoscopic techniques minimized complications and hastened a patient's return to full activities.^24,29^ When endoscopic techniques are not feasible, open curettage with or without bone augmentation and cannulated screw decompression do provide higher rates of healing and pain improvement than observation or injection.^16^

Although corticosteroid and bone marrow injections have been well researched in UBCs in long bones, our review of the literature showed that steroid or bone marrow injection in the calcaneus might not be optimal. Some studies of corticosteroid and bone marrow injection demonstrate increased healing rates and resolution of pain.^6,16,26^ However, other studies have shown that injections in the calcaneus are not a reliable treatment modality.^17^ This being the case, and with limited studies conducted in our population of interest, we recommend reserving corticosteroid and bone marrow injection for symptomatic patients who are not candidates for or are resistant to surgical intervention.

Because surgical intervention has well-documented healing and pain improvements with a very few complications reported, it should be preferred over injection when a patient is symptomatic and exceeds the critical size. Patients who are asymptomatic and do not meet the critical cyst size should be observed and reevaluated periodically for radiographic cyst progression and onset of symptoms. Symptomatic patients who do not meet critical size criteria and asymptomatic patients who do meet the critical size criteria should be treated at the discretion of the orthopaedic surgeon. Surgeons should weigh the risk of pathologic fracture, the risks of surgery, and the patient's overall functionality limited by pain.

With limited literature on managing simple bone cyst of the calcaneus in the pediatric population, more investigation is needed. Finally, small sample sizes and varied reporting methods may have led to bias within our study. Future studies are required to continue to test the validity of the Pogoda criteria of critical size and symptomatology. Long-term follow-up of patients who received endoscopic curettage with allograft is also needed to determine whether there is cyst recurrence or complication.
